# mRNA COVID-19 Vaccine Effectiveness Against Severe Outcomes Among Adults Hospitalized with COVID-19 from May 2021 to January 2023

**DOI:** 10.3390/vaccines14010045

**Published:** 2025-12-30

**Authors:** Gabriella Ess, Ashley M. Lew, Ashley Tippett, Luis W. Salazar, Chris Choi, Khalel De Castro, Elizabeth G. Taylor, Olivia D. Reese, Humerazehra Momin, Caroline R. Ciric, Amrita Banerjee, Amy Keane, Laura A. Puzniak, Robin Hubler, Srinivas Valluri, Benjamin Lopman, Nadine Rouphael, Satoshi Kamidani, John M. McLaughlin, Evan J. Anderson, Christina A. Rostad

**Affiliations:** 1 Department of Pediatrics, Emory University School of Medicine, Atlanta, GA 30322, USAashley.tippett@emory.edu (A.T.); luis.w.salazar@emory.edu (L.W.S.); tayloreg2@vcu.edu (E.G.T.); nroupha@emory.edu (N.R.);; 2 Pfizer Inc., Collegeville, PA 19426, USA; 3 Rollins School of Public Health, Emory University, Atlanta, GA 30322, USA; 4 The Hope Clinic of the Emory Vaccine Center, Division of Infectious Diseases, Department of Medicine, Emory University, Decatur, GA 30030, USA; 5 Children’s Healthcare of Atlanta, Atlanta, GA 30329, USA

**Keywords:** mRNA vaccination, severity, outcomes, hybrid immunity, hospitalization

## Abstract

**Background/Objectives**: COVID-19 mRNA vaccines protect against hospitalization, but less is known about real-world vaccine effectiveness (VE) against other severe outcomes. **Methods**: We enrolled adults hospitalized with acute respiratory illness at two hospitals in Atlanta, Georgia, USA from May 2021 to January 2023. Participants were eligible if they had standard-of-care COVID-19 testing or provided an upper respiratory swab for analysis. Vaccination status was confirmed through the state registry. mRNA COVID-19 VE among those with severe outcomes was determined using a test-negative case–control design with stepwise logistic regression adjusting for confounding variables. **Results**: Of 1973 participants eligible for analysis, 886 (44.9%) were unvaccinated, 641 (32.5%) received a primary series, and 446 (22.6%) received a primary series plus ≥ 1 booster. A total of 734 (37.2%) were positive for COVID-19. During the pre-Delta/Delta (2 May 2021–19 December 2021) vs. Omicron (20 December 2021–31 January 2023) eras, adjusted COVID-19 mRNA VE of a primary series compared to no vaccination was 85.5% (95% CI: 77.0%, 90.8%) vs. 38.2% (95% CI: 11.5%, 56.8%) overall, 90.0% (95% CI: 82.6%, 94.2%) vs. 54.4% (95% CI: 9.0%, 77.1%) among those with radiographic pneumonia, and 94.4% (95% CI: 80.5%, 98.4%) vs. 62.5% (95% CI: 19.0%, 82.7%) among those admitted to the ICU. VE against severe outcomes was highest within the 6 months following vaccination and during the pre-Delta/Delta era. A booster dose partially restored VE against Omicron-associated hospitalization and pneumonia. **Conclusions**: COVID-19 mRNA vaccines were effective at preventing hospitalization and other severe outcomes in adults during periods of pre-Delta/Delta and Omicron variant circulation.

## 1. Introduction

COVID-19 has been associated with significant morbidity and mortality since the onset of the pandemic in March 2020. As of May 2024, there were over 100 million COVID-19 cases and over 1.2 million total deaths associated with COVID-19 in the United States (US) [1]. Certain populations, including those who are immunocompromised or have other underlying comorbidities, are at a higher risk of severe COVID-19 [2,3,4]. COVID-19 vaccines have played an important role in protecting against infection, severe disease, and death due to COVID-19 [5,6].

Two mRNA-based vaccines for COVID-19, BNT162b2 (Pfizer Inc., New York, NY USA) and mRNA-1273 (Moderna, Inc. Cambridge, MA, USA), were made available initially through an Emergency Use Authorization (EUA) in December 2020, with full approval granted in August 2021 and January 2022, respectively [7,8]. Vaccine effectiveness (VE) against symptomatic COVID-19 was high for both vaccines in Phase III trials, at 95.0% for BNT162b2 and 94.1% for mRNA-1273 vaccine in SARS-CoV-2 naïve populations [9,10,11]. However, real-world VE decreased from the original estimates due to factors such as the emergence of SARS-CoV-2 variants, including Delta and Omicron with its subvariants [12,13,14]. Vaccine recommendations evolved over time to include the addition of booster doses in alignment with predominant circulating variants [15,16], and these recommendations continue to evolve. It is therefore critical to provide real-world evidence to inform decision-making about vaccine policies and recommendations. While it is known that COVID-19 vaccines offer protection against mortality, with COVID-19 vaccines preventing an estimated 20 million deaths worldwide during the first year of vaccine administration alone, additional information about VE against severe disease outcomes, including prolonged hospitalization and ICU admission, is important in the setting of waning immunity and emergence of new SARS-CoV-2 variants [17,18].

As the majority of the US population has now been exposed to COVID-19, hybrid immunity has become more common: the combination of infection- and vaccine-induced immunity. By the end of 2022, an estimated 96.7% (95% CI: 93.9%, 98.3%) of individuals of the US aged 16+ were seropositive for SARS-CoV-2, with 77.5% (95% CI: 75.1%, 79.8%) having evidence of infection-induced seropositivity [19]. Like immunity to COVID-19 from vaccination alone, hybrid immunity wanes over time from the last exposure to SARS-CoV-2, but prior research suggests that it may confer additional protection compared to prior infection alone and increased protection with increased doses of COVID-19 vaccines [20,21,22]. Nevertheless, the capacity of hybrid immunity to confer protection against severe COVID-19 outcomes is incompletely understood, in part due to the differential effects of infection timing and variant on disease outcomes [23,24].

Additionally, the effectiveness of COVID-19 vaccines against severe outcomes among racial and ethnic minority groups is incompletely understood. This is important because COVID-19 has caused disproportionate burden of disease among some groups during different waves of the pandemic. For example, a systemic review and meta-analysis of 4.3 million patients from 68 studies found that members of racial and ethnic minority groups, including African Americans, Hispanic, and Asian Americans had higher rates of COVID-19 positivity and disease severity than White populations [25]. Furthermore, people of Black race and Mixed races were found to have significantly lower rates of COVID-19 vaccination compared to White populations [26]. As COVID-19 vaccination has been found to reduce disparities in outcomes among certain groups [27], understanding VE among diverse populations could help inform vaccine recommendations.

This study aimed to estimate the effectiveness of a primary series and booster dose of mRNA COVID-19 vaccines and secondarily of hybrid immunity using a test-negative, case–control design among hospitalized participants and those experiencing other severe outcomes. The study was performed at two hospitals in Atlanta, Georgia, where there is a diverse patient population with a predominance of African American patients. This manuscript expands upon data previously published demonstrating the effectiveness of the BNT162b2 vaccine against hospitalization [28].

## 2. Materials and Methods

### 2.1. Participant Enrollment and Identification

For this test-negative, case–control study, participants were eligible for enrollment if they were ≥18 years of age, were hospitalized at one of two metropolitan Atlanta hospitals (Emory University Hospital, EUH; Emory University Hospital Midtown, EUHM) for acute respiratory infection (ARI) between May 2021 and January 2023 and had previously provided a nasopharyngeal (NP) or nasal swab as part of standard-of-care (SOC) testing or were willing to provide an NP/nasal swab specimen at the time of study enrollment and comply with all requested data collection. ARI was defined as having either ARI symptoms, including nasal congestion, rhinorrhea, sore throat, hoarseness, new or increased-from-baseline cough, sputum production, dyspnea, or wheezing, or having an admission diagnosis suggestive of ARI, including pneumonia, upper respiratory infection (URI), bronchitis, cough, asthma exacerbation, viral respiratory illness, and respiratory distress. Potential participants were excluded if they had received SARS-CoV-2 directed antiviral treatment within the preceding 30 days or if they had received COVID-19 monoclonal antibody therapy or COVID-19 convalescent serum within the preceding 90 days of NP or nasal swab collection.

Participant demographic, epidemiological, and clinical data were collected from a patient interview, when possible, and the electronic medical record (EMR). Data was stored in a REDCap database. Vaccination data were collected from the EMR and verified through the Georgia Immunization Registry (GRITS). Discrepancies between the EMR and GRITS were adjudicated by the study principal investigator and data manager. Participants were considered ‘Unvaccinated’ if they had not received any prior COVID-19 vaccination. Participants who had received the initially recommended primary series vaccination regimen were considered to have a primary series: 2 doses of any mRNA vaccine for immunocompetent patients, and 3 doses of any mRNA vaccine for immunocompromised patients. Patients were classified in the boosted group if they had received at least one additional dose of any mRNA vaccine after meeting the primary series eligibility. There was no minimum time interval requirement between doses for participants. Participants were considered immunocompromised if they had a history of cancer, HIV/AIDS, solid organ transplant, hematopoietic stem cell transplant, splenectomy, chronic steroid use, or rheumatologic conditions. A diagnosis of pneumonia was defined using radiographic reports from the medical chart. “Definite” pneumonia required clear notation in the radiologist’s report. If the radiologist report did not state pneumonia to be the clear and only diagnosis but included consolidation, pleural effusion (not “trace” or “possible”), or alveolar/interstitial infiltrates, or if the final diagnosis included pneumonia/infection plus one other etiology then participants were categorized as having “probable” pneumonia. These were captured in the database as a combined variable of definite/probable pneumonia. Participants were asked to report prior history of SARS-CoV-2 infection during the patient interview. Hybrid immunity was defined as receiving mRNA vaccination as described above and having history of SARS-CoV-2 infection noted in the medical chart. Self-reported prior infection data was used to inform time since prior infection estimates if the medical records did not provide adequate details. Participants who had received partial primary COVID-19 vaccination schedules, those who had received a non-mRNA COVID-19 vaccine, and those who had received COVID-19 vaccination within 14 days of hospital admission were excluded from this analysis.

The study was reviewed and approved by the Institutional Review Board at Emory University, and written informed consent was obtained from eligible participants or their legally authorized representatives as applicable.

### 2.2. COVID-19 Testing

Participants were tested for respiratory pathogens by a standard-of-care (SOC) molecular test, typically either GeneXpert (Cepheid, Sunnyvale, CA, USA) or BioFire^®^ (Salt Lake City, UT, USA) respiratory viral panel (RVP) tests, or study-specific testing [29]. Study-specific testing used combined NP/oropharyngeal (OP) swabs with a BioFire^®^ RVP.

### 2.3. Statistical Analyses

COVID-19 cases were defined as participants who tested positive for COVID-19 by any source, whether SOC molecular testing or the BioFire^®^ RVP, within 14 days prior to enrollment. Controls were defined as those negative for COVID-19 by all testing sources and methods. Potentially eligible participants whose COVID-19 swab was collected greater than 72 h after admission were excluded from the study to avoid potential nosocomial infections. Although the incubation period for nosocomial infections varies by SARS-CoV-2 variant, it typically ranges from 3 to 6 days [30]. Sociodemographic and clinical characteristics were compared using bivariate analyses. T-tests, Chi-squared, or Fisher exact tests were used as appropriate. A *p*-value ≤ 0.05 was considered statistically significant when comparing cases vs. controls. Bonferroni correction was applied when comparing unvaccinated vs. primary series and vs. booster dose to reduce the risk of type I errors in the setting of multiple bivariate comparisons; these results were considered statistically significant with *p*-values ≤ 0.025. Absolute VE compared to no vaccination was estimated with a test-negative, case–control design using the formula VE = (1 − odds ratio (OR)) × 100%, where OR was defined as the odds of COVID-19 vaccination (primary series or boosted) among COVID-19-positive cases divided by the odds of vaccination among COVID-19-negative controls. As a secondary analysis, to assess the impact of hybrid immunity, the exposure status of cases and controls were stratified by any self-reported history of infection by comparing hybrid immunity to unvaccinated individuals without a history of prior infection. Stepwise multivariate logistic regression was performed to find the adjusted COVID-19 mRNA VE, with a model inclusion criterion of 0.05. ORs were adjusted for age, enrollment quarter (i.e., calendar time by 3-month intervals), sex, race/ethnicity, immunocompromised status, and enrollment site. Severe outcomes included hospitalization, duration of hospitalization ≥ 4 days, definite/probable pneumonia, ICU admission, mechanical ventilation, and death. Four days was chosen as the cutoff duration for severe hospitalization, as it was the median duration of hospitalization among the cohort. VE estimates for the pre-Delta/Delta variant-predominant and Omicron variant-predominant eras were calculated by restricting the analyses to participants admitted during those periods (2 May 2021 to 19 December 2021, and 20 December 2021 to 31 January 2023, respectively). All analyses were performed using SAS v9.4 (SAS Institute Inc, Cary, NC, USA).

## 3. Results

### 3.1. Study Population

**Figure 1:**
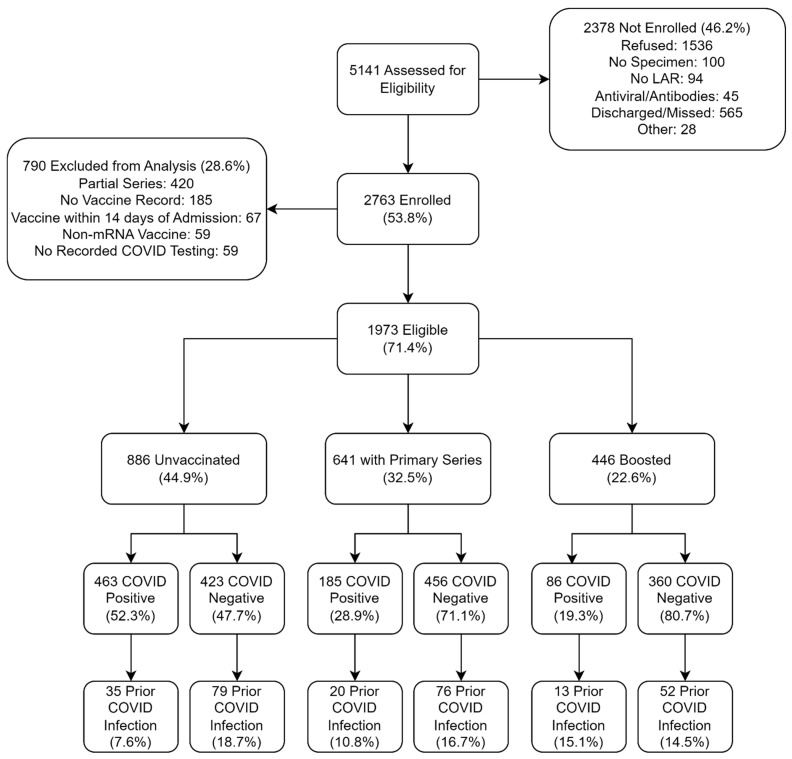
Consort Diagram of participants by mRNA COVID-19 vaccination status and SARS-CoV-2 Infection Status. Note that the primary series and boosted groups are mutually exclusive. LAR, legally authorized representative.

Of 5141 eligible patients, 2763 were enrolled (53.8%) (Figure 1). The demographic characteristics of enrolled and non-enrolled patients are shown in Supplementary Table S1. 790 participants (28.6%) were excluded from this analysis due to either receipt of a partial series of mRNA vaccine (*n* = 420), not having a vaccine record (*n* = 185), receipt of a COVID-19 vaccine within 14 days of admission (*n* = 67), receipt of a non-mRNA COVID-19 vaccine (*n* = 59), or no recorded COVID-19 testing (*n* = 59) (Figure 1). Of the 1973 remaining participants, 886 (44.9%) were unvaccinated, 641 (32.5%) received the primary series, and 446 (22.6%) received ≥ 1 booster dose. The distribution of homologous and heterologous vaccine schedules is shown in the Supplementary Table S2. The median time from the most recent dose of vaccine to hospital admission was 259 days [Interquartile Range (IQR): 143, 385] for participants who received a primary series and 153 days [IQR: 75, 274] for participants who received ≥ 1 booster dose. 1239 participants (62.8%) tested negative for SARS-CoV-2 (‘Controls’), and 734 (37.2%) tested positive (‘Cases’) (Figure 1). The distribution of other pathogens detected is shown in the Supplementary Table S3.

COVID-19 cases were younger than controls (median age 56 vs. 61 years, *p* < 0.0001), were admitted earlier in the study period than controls (*p* < 0.0001) and were less commonly admitted during the Omicron-predominant period (51.9% vs. 80.9%, *p* < 0.0001) (Table 1). Cases more commonly had children <18 years residing at home (30.1% vs. 19.6%, *p* < 0.0001), were more commonly employed (39.6% vs. 21.0%, *p* < 0.0001), were more commonly adherent to social distancing and masking guidance during the time period, had more commonly traveled in the month prior to enrollment (17.0% vs. 8.8%, *p* < 0.0001), and less commonly reported current or prior smoking (*p* < 0.0001) (Table 2). Cases also had a higher body mass index (BMI) (median 29.8 vs. 28.1, *p* = 0.014) but otherwise were generally healthier than controls and less commonly had a medical history of chronic respiratory disease, blood disorders, an immunocompromising condition, cardiac disease, or stroke. COVID-19 cases were more commonly pregnant than controls (3.1% vs. 1.7%, *p* = 0.036) (Table 3). In terms of disease severity, cases less commonly required CPAP/BIPAP (8.6% vs. 11.5%, *p* = 0.038) but more commonly had definite/probable radiographic pneumonia (59.7% vs. 47.2%, *p* < 0.0001) and duration of hospitalization ≥ 4 days (54.9% vs. 50.2%, *p* = 0.043) (Table 3).

Participants vaccinated with a primary series and boosted participants were older than unvaccinated participants (median age 63 and 69 vs. 52, *p* < 0.0001), were less commonly Black race (58.7% and 47.5% vs. 72.7%, *p* < 0.0001), were admitted later in the study period and were more commonly admitted during the Omicron-predominant period (76.4% and 98.0% vs. 51.6%, *p* < 0.0001) (Table 1). Participants with a primary series and boosted participants less commonly had children <18 residing in the home (16.1% and 13.7% vs. 33.9%, *p* < 0.0001), were less commonly employed (25.1% and 17.3% vs. 35.3%, *p* < 0.0001), more commonly reported past, non-current smoking history (33.1% and 36.1% vs. 26.2%, *p* < 0.0001), and had lower basic and instrumental activities of daily living scores (*p* < 0.0001) (Table 2). Primary series and booster vaccinated participants had lower BMIs (median 28.2 and 28.2 vs. 29.3, *p* = 0.033 and *p* = 0.010), more commonly had history of chronic respiratory disease, immunosuppressive conditions, cardiac disease, chronic kidney disease, dementia, diabetes, stroke, and home oxygen use. In terms of severity, they less often developed definite/probable radiographic pneumonia (52.6% and 38.1% vs. 58.2%, *p* = 0.028 and *p* < 0.0001) and less commonly had duration of hospitalization ≥ 4 days (49.5% and 49.1% vs. 55.2%, *p* = 0.027 and 0.036), although these results were not all statistically significant (Table 3).

### 3.2. Vaccine Effectiveness Analyses

After controlling for age, enrollment quarter (i.e., calendar time by 3-month intervals), sex, race/ethnicity, immunocompromised status, and enrollment site, VE among hospitalized participants admitted during the pre-Delta/Delta variant predominant period was 85.5% (95% CI: 77.0%, 90.8%) for an mRNA COVID-19 vaccine primary series and 96.4% (95% CI: 68.9%, 99.6%) for ≥1 booster (Table 4). For participants admitted to the hospital during the Omicron-predominant period, adjusted VE among hospitalized participants was 38.2% (95% CI: 11.5%, 56.8%) for a primary series and 48.5% (95% CI: 20.8%, 66.5%) for those with ≥1 booster dose.

In terms of other severe outcomes, for participants admitted during the pre-Delta/Delta era, the adjusted VE of an mRNA COVID-19 vaccine primary series was 90.0% (95% CI: 82.6%, 94.2%) against definite/probable radiographic pneumonia, 83.6% (95% CI: 68.4%, 91.5%) against duration of hospitalization ≥ 4 days, and 94.4% (95% CI: 80.5%, 94.4%) against ICU admission. We were unable to calculate VE of ≥1 booster dose during the pre-Delta/Delta era due to the small number of participants who received a booster dose during this period. For participants admitted during the Omicron era, the adjusted VE of an mRNA COVID-19 vaccine primary series was 42.9% (95% CI: 2.3%, 66.7%) against definite/probable radiographic pneumonia, 49.7% (95% CI: 16.3%, 69.8%) against duration of hospitalization ≥ 4 days, and 62.5% (95% CI: 19.0%, 82.7%) against ICU admission. A booster dose partially restored VE against severe outcomes during the Omicron era. The adjusted VE of ≥1 booster dose of an mRNA COVID-19 vaccine was 54.4% (95% CI: 9.0%, 77.1%) against definite/probable radiographic pneumonia, 57.4% (95% CI: 20.6%, 77.2%) against duration of hospitalization ≥ 4 days, and 48.7% (95% CI: −40.9%, 81.4%) against ICU admission.

Overall, VE was generally higher during the pre-Delta/Delta era than the Omicron era (Table 4). When stratified by time since vaccination (<6 months vs. ≥6 months) (Table 4), VE estimates were generally higher within 6 months of vaccination. When stratified by age group (18–64 years and ≥65 years) (Supplementary Table S4), VE estimates were generally higher among older adults. VE estimates against more rare severe outcomes (i.e., mechanical ventilation and death) were not evaluable due to small number of participants with these outcomes in the study (Supplementary Table S5).

As a secondary analysis, we determined the effectiveness of hybrid immunity against the same severe outcomes and hypothesized that the additional antigen exposure from natural infection would be associated with higher levels of protection. For participants admitted during the pre-Delta/Delta era, the adjusted effectiveness of an mRNA COVID-19 vaccine primary series plus self-reported history of COVID-19 when compared to unvaccinated participants without history of COVID-19 was 92.8% (95% CI: 63.5%, 98.6%) against COVID-19 associated hospitalization and 92.7% (95% CI: 60.6%, 98.6%) against definite/probable radiographic pneumonia, while hybrid immunity effectiveness against duration of hospitalization ≥ 4 days was not statistically significant and against ICU admission was not evaluable due to sample size (Table 5). Effectiveness of hybrid immunity with ≥1 booster dose was not evaluable during the pre-Delta/Delta era (against all severe outcomes) or during the Omicron era (against ICU admission, mechanical ventilation, or death) due to the small number of participants with hybrid immunity and these outcomes. For participants admitted during the Omicron era, point estimates of adjusted VE of an mRNA COVID-19 vaccine primary series and ≥1 booster dose against COVID-19 associated hospitalization, radiographic pneumonia, and duration of hospitalization ≥ 4 days were positive, but not statistically significant.

VE power calculations are shown in Supplementary Tables S6–S8. Based on these, the study was underpowered to ascertain VE against some of the stratified outcomes, including VE of booster doses during the Pre-Delta/Delta era (due to insufficient booster vaccination coverage) and effectiveness of hybrid immunity during the Omicron era (due to lower numbers of cases and lower effectiveness against severe outcomes).

## 4. Discussion

mRNA COVID-19 vaccines were effective against hospitalization and severe outcomes among adults hospitalized for acute respiratory illness at two hospitals in Atlanta, Georgia with a predominantly African American population from May 2021 to January 2023. VE was greatest during the pre-Delta/Delta era and within 6 months of vaccination. The primary series alone and with ≥1 booster vaccine provided significant protection from definite/probable radiographic pneumonia, duration of hospitalization ≥ 4 days, and ICU admission. Because almost half (44.9%) of the study population was unvaccinated at enrollment, opportunity existed for increasing vaccine coverage to our diverse study population to reduce the risk of COVID-19 related hospitalization and severe outcomes during the study period.

Our pre-Delta/Delta estimates of mRNA COVID-19 VE among hospitalized participants were high and comparable to other studies during the early pandemic according to a systematic literature review [31]. A booster dose almost completely protected against hospitalization during the pre-Delta/Delta era, also consistent with prior studies [32]. Our VE estimates of the primary series and ≥1 booster dose decreased during the Omicron era, as has been previously described [32,33], and were likely attributable in part to immune evasion by this variant [34]. Many studies have found higher VE of a booster dose when compared with a primary series alone. However, a systematic review of the effectiveness of pre-Omicron COVID-19 vaccines against the Omicron variant in comparison to Delta found reduced VE against Omicron-associated hospitalization and severe outcomes that was only partially boostable and quickly waned [35]. Multiple factors may modify booster dose VE, including the prime and boosting strains, the time interval since the most recent booster dose, and the currently circulating variants and their potential to evade pre-existing immunity [36]. Immunologic imprinting is thought to play a key role in individual responses to novel variants, as the initial exposing strains direct subsequent responses to slightly different antigens encountered in the context of B cell memory [37]. In our study, VE of ≥1 booster dose was higher against most evaluable severe outcomes than the primary series alone within both the pre-Delta/Delta and Omicron eras; however, VE of ≥1 booster dose notably waned and was no longer statistically significant >6 months after vaccination during the Omicron era. While we were unable to calculate a statistically significant VE for COVID-19 associated death due to the small number of observed events in our study population, larger studies have found significant protection against mortality [32,38].

In terms of hybrid immunity, previous studies have found a higher, more durable effectiveness of hybrid immunity compared to COVID-19 vaccination alone [39]. Hybrid immunity has been found to increase mucosal immunity [40] and to broaden humoral [41] and cellular responses [42]. A post hoc cross-protocol analysis of Moderna, AstraZeneca, Janssen, and Novavax phase 3 COVID-19 vaccine clinical trials found that hybrid immunity provided near-complete protection against severe disease across platforms [21]. In our study, we found that hybrid immunity was effective among those who had received a primary series and had disease onset during the pre-Delta/Delta era; however, limitations in sample size during the Omicron era precluded our ability to draw firm conclusions about hybrid immunity in that era. Point estimates for effectiveness of hybrid immunity among those who had additionally received a booster dose and among those enrolled during the Omicron era were positive, but not statistically significant. This lack of observed significant effectiveness may have been attributable to limitations in sample size or to misclassification of subclinical infections due to recall bias. Another possibility is that prior infection had less impact among those who were infected with a different variant due to immune imprinting. The need to overcome immune imprinting in order to confer protection to emerging variants coinciding with waning pre-existing immunity underscores the need for revaccination [43], which remains the cornerstone to protect against severe COVID-19 and to mitigate future outbreaks.

In terms of public policy recommendations, the Advisory Committee for Immunization Practices (ACIP) to the Centers for Disease Control and Prevention (CDC) has historically made immunization recommendations based on a scientific evidence to recommendation (EtR) framework [44]. This framework has included the critical assessment of the public health importance of a disease, the risks and benefits of vaccination, the values and acceptability among the affected population, resource utilization, equity, feasibility, and finally, the balance of consequences. Real-world safety and effectiveness analyses have been iterative and ongoing. Thus, there has not been a VE “threshold” that alone has been clinically meaningful. Rather, VE in the context of the above domains and the public policy goals have guided vaccine recommendations. For COVID-19, a public policy goal has been to reduce hospitalizations and other severe disease manifestations. Pooled VE estimates of the 2023–2024 and bivalent formulations used to inform the 2024–2025 EtRs showed pooled VE of 44% (95% CI 34–52%) against COVID-19 associated hospitalizations in adolescents and adults and 23% (95% CI 8–36%) against COVID-19 associated deaths [45]. While we did not find statistically significant VE against mortality, our VE estimates against other severe outcomes during the Omicron era were generally similar to those of the pooled EtR analyses, albeit with waning after 6 months. Thus our findings align with and support the recommendation for COVID-19 primary series and booster in the era of Omicron circulation to prevent severe disease.

This study has limitations. A large proportion of potentially eligible individuals did not enroll, and more were excluded based on vaccine history, which may have led to selection bias that differed by group. For example, if non-enrolled patients were sicker and less commonly vaccinated, this may have led to an underestimation of VE against severe outcomes. Potentially eligible participants whose COVID-19 swab was collected greater than 72 h after admission were excluded from the study to avoid potential nosocomial infections, but this may have also led to a selection bias. There were some baseline differences between cases and controls, such as adherence to social distancing behaviors, that could have impacted COVID-19 exposure and VE estimates.

We adjusted for confounding using step-wise multivariable logistic regression; however, this type of model is prone to overfitting and inflated type I errors. Some VE estimates, particularly for outcomes like death or mechanical ventilation, were unstable due to insufficient sample size. Further, the broad definition of definite/probable pneumonia may have led to misclassification of incidental radiographic findings unrelated to clinical pneumonia. There was likely overlap in the severe outcomes participants experienced, and thus the VE estimates for each outcome were not independent from each other. The study period spanned two distinct eras with different vaccination rates, variant circulation, and baseline immunity. While we adjusted VE estimates for calendar time by quarter, there may have been residual impact of temporal confounding. This analysis was completed prior to the emergence of newer Omicron sub-variants, such as the JN.1 lineage, and future research is therefore needed to assess VE against these and other variants with updated antigen-specific monovalent vaccines. In addition, all diagnoses of prior COVID-19 may not have been accurately captured due to recall bias in the interview, if a participant interview was not possible, if prior infections were mild or asymptomatic, or lack of prior diagnostic testing. These limitations may have impacted our ability to assess hybrid immunity, and future research is needed to better understand the impact of hybrid immunity in preventing severe outcomes. The sample size was insufficient to directly compare vaccine types (mRNA-1273 vs. BNT162b2) or booster antigen specificities (prototype vs. bivalent BA.4/5), calculate VE for different strains of SARS-CoV-2 beyond VE estimates during periods of variant predominance, or calculate VE amongst participants with certain severe outcomes, although important differences may exist. In addition, a test-negative, case–control design has a few inherent limitations, such as the impact of vaccination or COVID-19 status misclassification errors on VE estimates and the assumption that COVID-19 vaccination does not affect the incidence of COVID-19-like illness [46,47,48]. Lastly, as this study occurred at only two sites within the same hospital system, the results may not be generalizable to all healthcare settings.

## 5. Conclusions

In conclusion, using prospective active surveillance and a test-negative, case–control design, we found that mRNA COVID-19 vaccination provided significant protection among participants experiencing severe outcomes, including COVID-19 associated hospitalization, pneumonia, hospitalization duration ≥ 4 days, and ICU admission among a predominantly African American study population in Atlanta, Georgia. These results provide further evidence of the benefits vaccines have had in the prevention of severe COVID-19 through the pre-Delta/Delta and Omicron eras. Future research should continue to investigate the real-world effectiveness of variant-specific COVID-19 vaccines in the setting of newly emerging SARS-CoV-2 variants.

## Figures and Tables

**Table 1:** Demographic Characteristics of Study Participants by SARS-CoV-2 Infection and Vaccination Status.

	Controls: SARS-CoV-2 Negative (*n* = 1239)	Cases: SARS-CoV-2 Positive (*n* = 734)	*p*-Value	Never Vaccinated for COVID-19 (*n* = 886)	Primary Series (*n* = 641)	*p*-Value (vs. Never Vaccinated)	≥1 Booster Dose (*n* = 446)	*p*-Value (vs. Never Vaccinated)
**Age, median [IQR], years**	61 [49, 71]	56 [43, 67]	**<0.0001**	52 [38, 62]	63 [53, 72]	**<0.0001**	69 [59, 76]	**<0.0001**
**Age by category, No. (%), years**			**<0.0001**			**<0.0001**		**<0.0001**
18–49	318 (25.7)	260 (35.4)		398 (44.9)	130 (20.3)		50 (11.2)	
50–64	414 (33.4)	238 (32.4)		312 (35.2)	220 (34.3)		120 (26.9)	
65–74	278 (22.4)	133 (18.1)		116 (13.1)	157 (24.5)		138 (30.9)	
≥75	229 (18.5)	103 (14.0)		60 (6.8)	134 (20.9)		138 (30.9)	
**Female sex, No. (%)**	686 (55.3)	418 (56.9)	0.494	519 (58.6)	346 (54.0)	0.07	239 (53.6)	0.083
**Race, No. (%)**			**0.013**			**<0.0001**		**<0.0001**
Black	743 (60.0)	489 (66.6)		644 (72.7)	376 (58.7)		212 (47.5)	
White	326 (26.3)	175 (23.8)		158 (17.8)	190 (29.6)		153 (34.3)	
Multiracial	31 (2.5)	18 (2.5)		21 (2.4)	13 (2.0)		15 (3.4)	
Other	22 (1.8)	8 (1.1)		12 (1.4)	10 (1.6)		8 (1.8)	
Not Specified	117 (9.4)	44 (6.0)		51 (5.8)	52 (8.1)		58 (13.0)	
**Ethnicity, No. (%)**			**0.005**			**0.007**		**<0.0001**
Non-Hispanic	1050 (84.7)	645 (87.9)		773 (87.2)	564 (88.0)		358 (80.3)	
Hispanic	34 (2.7)	29 (4.0)		41 (4.6)	12 (1.9)		10 (2.2)	
Not Specified	155 (12.5)	60 (8.2)		72 (8.1)	65 (10.1)		78 (17.5)	
**Insurance, No. (%)**			**<0.0001**			**<0.0001**		**<0.0001**
Public	520 (42.0)	299 (40.7)		379 (42.8)	279 (43.5)		161 (36.1)	
Private	243 (19.6)	233 (31.7)		243 (27.4)	154 (24.0)		79 (17.7)	
Multiple	377 (30.4)	137 (18.7)		144 (16.3)	178 (27.8)		192 (43.0)	
None	97 (7.8)	63 (8.6)		117 (13.2)	30 (4.7)		13 (2.9)	
Other/Unknown	2 (0.2)	2 (0.3)		3 (0.3)	0 (0.0)		1 (0.2)	
**Site of Enrollment, No. (%)**			**0.04**			**<0.0001**		**<0.0001**
Hospital 1	571 (46.1)	374 (51.0)		367 (41.4)	332 (51.8)		246 (55.2)	
Hospital 2	668 (53.9)	360 (49.0)		519 (58.6)	309 (48.2)		200 (44.8)	
**Month of Admission, No. (%)**			**<0.0001**			**<0.0001**		**<0.0001**
MAY 21–JUL 21	87 (7.0)	91 (12.4)		135 (15.2)	43 (6.7)		0 (0.0)	
AUG 21–OCT 21	82 (6.6)	228 (31.1)		239 (27.0)	68 (10.6)		3 (0.7)	
NOV 21–JAN 22	99 (8.0)	150 (20.4)		127 (14.3)	104 (16.2)		18 (4.0)	
FEB 22–APR 22	133 (10.7)	34 (4.6)		59 (6.7)	73 (11.4)		35 (7.9)	
MAY 22–JUL 22	90 (7.3)	72 (9.8)		58 (6.5)	61 (9.5)		43 (9.6)	
AUG 22–OCT 22	339 (27.4)	67 (9.1)		118 (13.3)	142 (22.1)		144 (32.3)	
NOV 22–JAN 23	409 (33.0)	92 (12.5)		150 (16.9)	150 (23.4)		203 (45.5)	
**Variant Period, No. (%)**			**<0.0001**			**<0.0001**		**<0.0001**
Pre-Delta/Delta	237 (19.1)	353 (48.1)		429 (48.4)	152 (23.7)		9 (2.0)	
Omicron	1002 (80.9)	381 (51.9)		457 (51.6)	489 (76.4)		437 (98.0)	
**Education level, No. (%)**			**0.0002**			**<0.0001**		**<0.0001**
<High School Grad/GED	160 (12.9)	71 (9.7)		124 (14.0)	64 (10.0)		43 (9.6)	
High School Grad/GED	346 (27.9)	183 (24.9)		276 (31.2)	158 (24.6)		95 (21.3)	
Some College/Associate degree/Trade School	274 (22.1)	209 (28.5)		226 (25.5)	159 (24.8)		98 (22)	
College Graduate	226 (18.2)	162 (22.1)		158 (17.8)	137 (21.4)		93 (20.9)	
Advanced Degree	113 (9.1)	64 (8.4)		47 (5.3)	71 (11.1)		59 (13.2)	
Unknown	120 (9.7)	45 (6.1)		55 (6.2)	52 (8.1)		58 (13)	
**Residence, No. (%)**			**0.003**			**0.002**		**<0.0001**
Private Home	1048 (84.6)	653 (89.0)		791 (89.3)	558 (87.1)		352 (78.9)	
SNF/Assisted Living/LTCF	29 (2.3)	17 (2.3)		8 (0.9)	18 (2.8)		20 (4.5)	
Other	46 (3.7)	29 (4/0)		41 (4.6)	17 (2.7)		17 (3.8)	
Unknown	116 (9.4)	35 (4.8)		46 (5.2)	48 (7.5)		57 (12.8)	

Abbreviations: IQR = Interquartile Range, SNF = Skilled Nursing Facility, LTCF = Long-term Care Facility. Bold *p*-values denote statistical significance.

**Table 2:** Social Factors/Behaviors of Study Participants by SARS-CoV-2 Infection and Vaccination Status.

	Controls: SARS-CoV-2 Negative (*n* = 1239)	Cases: SARS-CoV-2 Positive (*n* = 734)	*p*-Value *	Never Vaccinated for COVID-19 (*n* = 886)	Primary Series (*n* = 641)	*p*-Value ** (vs. Never Vaccinated)	≥1 Booster Dose (*n* = 446)	*p*-Value ** (vs. Never Vaccinated)
**Any Children age <18 years at home, No. (%)**			**<0.0001**			**<0.0001**		**<0.0001**
None	885 (71.4)	477 (65.0)		543 (61.3)	490 (76.4)		329 (73.8)	
≥1	243 (19.6)	221 (30.1)		300 (33.9)	103 (16.1)		61 (13.7)	
Unknown	111 (9.0)	36 (4.9)		43 (4.9)	48 (7.5)		56 (12.6)	
**Children aged <5 years at home, No. (%)**			**<0.0001**			**<0.0001**		**<0.0001**
None	1027 (82.9)	601 (81.9)		707 (79.8)	558 (87.1)		363 (81.4)	
≥1	101 (8.2)	97 (13.2)		136 (15.3)	35 (5.5)		27 (6.1)	
Unknown	111 (9.0)	36 (4.9)		43 (4.9)	48 (7.5)		56 (12.6)	
**Children aged 5–17 years at home, No. (%)**			**<0.0001**			**<0.0001**		**<0.0001**
None	931 (75.1)	524 (71.4)		599 (67.6)	512 (79.9)		344 (77.1)	
≥1	197 (15.9)	174 (23.7)		244 (27.5)	81 (12.6)		46 (10.3)	
Unknown	111 (9)	36 (4.9)		43 (4.9)	48 (7.5)		56 (12.6)	
**Employment**			**<0.0001**			**<0.0001**		**<0.0001**
Yes	260 (21.0)	291 (39.6)		313 (35.3)	161 (25.1)		77 (17.3)	
Employed as HCW			0.451			0.080		0.122
Yes	31 (11.9)	41 (14.1)		33 (10.5)	26 (16.2)		13 (16.9)	
No	229 (88.1)	250 (85.9)		280 (89.5)	135 (83.8)		64 (83.1)	
Work Location, No. (%)			0.077			0.078		**0.009**
Always leave home for work	156 (60.0)	184 (63.2)		205 (65.5)	96 (59.6)		39 (50.5)	
Leave home for work most of the time	16 (6.2)	22 (7.6)		16 (5.1)	13 (8.1)		9 (11.7)	
Occasionally leave home for work	35 (13.5)	20 (6.9)		22 (7.9)	21 (13)		12 (15.6)	
Exclusively work from home	53 (20.4)	65 (22.3)		70 (22.4)	31 (19.3)		17 (22.1)	
Face-to-face contact with public			0.117			**0.003**		0.132
Yes	154 (59.2)	153 (52.6)		157 (50.2)	104 (64.6)		46 (59.7)	
No	106 (40.8)	138 (47.4)		156 (49.8)	57 (35.4)		31 (40.3)	
No	573 (46.2)	265 (36.1)		329 (37.1)	288 (44.9)		221 (49.6)	
N/A (Disability)	295 (23.8)	144 (19.6)		201 (22.7)	146 (22.8)		92 (20.6)	
Unknown	111 (9.0)	34 (4.6)		43 (4.9)	46 (7.2)		56 (12.6)	
**Retired, No. (%)**			**<0.0001**			**<0.0001**		**<0.0001**
Yes	393 (31.7)	173 (23.6)		148 (16.7)	224 (34.9)		194 (43.5)	
No	437 (35.3)	386 (52.6)		493 (55.6)	224 (34.9)		106 (23.8)	
N/A (Unemployed)	298 (24.0)	141 (19.2)		202 (22.8)	147 (22.9)		90 (20.2)	
Unknown	111 (9.0)	34 (4.6)		43 (4.9)	46 (7.2)		56 (12.6)	
**Adherence to Social Distancing, No. (%)**			**<0.0001**			0.230		**<0.0001**
Always	677 (54.6)	465 (63.4)		515 (58.1)	371 (57.9)		256 (57.4)	
Usually	192 (15.5)	135 (18.4)		155 (17.5)	116 (18.1)		56 (12.6)	
About half the time	124 (10.0)	49 (6.7)		79 (8.9)	54 (8.4)		40 (9.0)	
Seldom/Never	134 (10.8)	50 (6.8)		93 (10.5)	53 (8.3)		38 (8.5)	
Unknown	112 (9.0)	35 (4.8)		44 (5.0)	47 (7.3)		56 (12.6)	
**Adherence to Masking in Public (indoors), No. (%)**			**<0.0001**			0.055		**<0.0001**
Always	684 (55.2)	492 (67.0)		533 (60.2)	390 (60.8)		253 (56.7)	
Usually	165 (13.3)	105 (14.3)		131 (14.8)	83 (12.9)		56 (12.6)	
About half the time	94 (7.6)	47 (6.4)		59 (6.7)	55 (8.6)		27 (6.1)	
Seldom/Never	184 (14.9)	55 (7.5)		119 (13.4)	66 (10.3)		54 (12.1)	
Unknown	112 (9.0)	35 (4.8)		44 (5.0)	47 (7.3)		56 (12.6)	
**Adherence to Masking in Public (outdoors), No. (%)**			**<0.0001**			0.211		**<0.0001**
Always	547 (44.1)	411 (56.0)		463 (52.3)	312 (48.7)		183 (41.0)	
Usually	128 (10.3)	93 (12.7)		103 (11.6)	76 (11.9)		42 (9.4)	
About half the time	107 (8.6)	66 (9.0)		69 (7.8)	61 (9.5)		43 (9.6)	
Seldom/Never	345 (27.8)	129 (17.6)		207 (23.4)	145 (22.6)		122 (27.4)	
Unknown	112 (9.0)	35 (4.8)		44 (5.0)	47 (7.3)		56 (12.6)	
**Out of state travel in past month, No. (%)**			**<0.0001**			0.131		**<0.0001**
None	1018 (82.2)	574 (78.2)		731 (82.5)	522 (81.4)		339 (76.0)	
Domestic/International	109 (8.8)	125 (17.0)		111 (12.5)	72 (11.2)		51 (11.4)	
Unknown	112 (9.0)	35 (4.8)		44 (5.0)	47 (7.3)		56 (12.6)	
**Smoking, No. (%)**			**<0.0001**			**<0.0001**		**<0.0001**
None	523 (42.2)	435 (59.3)		445 (50.2)	317 (49.5)		196 (43.9)	
Current	184 (14.9)	62 (8.4)		155 (17.5)	58 (9.0)		33 (7.4)	
Past	412 (33.3)	193 (26.3)		232 (26.2)	212 (33.1)		161 (36.1)	
Unknown	120 (9.7)	44 (6.0)		54 (6.1)	54 (8.4)		56 (12.6)	
**Drink Alcohol, No. (%)**			**0.001**			0.125		**<0.0001**
Never	722 (58.3)	443 (60.4)		519 (58.6)	380 (59.3)		266 (59.6)	
Monthly or Less	267 (21.5)	167 (22.8)		203 (22.9)	149 (23.2)		82 (18.4)	
2–4 times/month	51 (4.1)	49 (6.7)		56 (6.3)	28 (4.4)		16 (3.6)	
2–3 times/week	53 (4.3)	22 (3.0)		41 (4.6)	20 (3.1)		14 (3.1)	
≥4 times/week	35 (2.8)	18 (2.5)		24 (2.7)	17 (2.7)		12 (2.7)	
Unknown	111 (9.0)	35 (4.8)		43 (4.9)	47 (7.3)		56 (12.6)	
**Activities of Daily Living (ADL) Scores, median [IQR]**								
Basic ADL (range, 0–6)	6 [6, 6]	6 [6, 6]	**<0.0001**	6 [6, 6]	6 [6, 6]	**0.022**	6 [6, 6]	**0.006**
Instrumental ADL								
Male & Female (range, 0–8)	8 [7, 8]	8 [8, 8]	**<0.0001**	8 [8, 8]	8 [7, 8]	**<0.0001**	8 [6, 8]	**<0.0001**
Male (range, 0–5)	5 [4.8, 5]	5 [5, 5]	**0.006**	5 [5, 5]	5 [5, 5]	**0.006**	5 [4, 5]	0.0495
Female (range, 0–8)	8 [6, 8]	8 [8, 8]	**<0.0001**	8 [8, 8]	8 [7, 8]	**0.005**	8 [6, 8]	**0.001**

* For comparisons of cases vs. controls, *p*-values ≤ 0.05 were considered statistically significant. ** For comparisons of unvaccinated vs. primary series or vs. booster series, Bonferroni correction was applied due to multiple bivariate comparisons, and *p*-values ≤ 0.025 were considered statistically significant. Bold *p*-values denote statistical significance. HCW, Healthcare worker.

**Table 3:** Clinical Characteristics of Study Participants by SARS-CoV-2 Infection and Vaccination Status.

	Controls: SARS-CoV-2 Negative (*n* = 1239)	Cases: SARS-CoV-2 Positive (*n* = 734)	*p*-Value *	Never Vaccinated for COVID-19 (*n* = 886)	Primary Series (*n* = 641)	*p*-Value ** (vs. Never Vaccinated)	≥1 Booster Dose (*n* = 446)	*p*-Value ** (vs. Never Vaccinated)
**BMI, median [IQR]**	28.1 [23.2, 34.8]	29.8 [24.6, 36.3]	**0.014**	29.3 [23.8, 36.3]	28.2 [23.5, 35.0]	0.033	28.2 [24.0, 34.1]	**0.010**
**BMI by category ^a^, No. (%)**			**<0.0001**			0.072		**0.016**
Underweight (<18.5)	69 (5.6)	18 (2.5)		34 (3.8)	39 (6.1)		14 (3.1)	
Healthy (18.5–24.9)	333 (26.9)	174 (23.7)		227 (25.6)	162 (25.3)		118 (26.5)	
Overweight (25.0–29.9)	328 (26.5)	174 (23.7)		205 (23.1)	161 (25.1)		136 (30.5)	
Obese, class 1 (30.0–34.9)	201 (16.2)	140 (19.1)		153 (17.3)	116 (18.1)		72 (16.1)	
Obese, class 2–3 (≥35.0)	301 (24.3)	206 (28.1)		247 (27.9)	157 (24.5)		103 (23.1)	
Unknown	7 (0.6)	22 (3.0)		20 (2.3)	6 (0.9)		3 (0.7)	
**Chronic Conditions, No. (%)**								
Chronic Respiratory Disease	528 (42.6)	188 (25.6)	**<0.0001**	280 (31.6)	257 (40.1)	**0.0006**	179 (40.1)	**0.002**
Chronic Obstructive Pulmonary Disease	323 (26.1)	94 (12.8)	**<0.0001**	140 (15.8)	155 (24.2)	**<0.0001**	122 (27.4)	**<0.0001**
Use oxygen supplementation at home	180 (14.5)	33 (4.5)	**<0.0001**	67 (7.6)	82 (12.8)	**0.0007**	64 (14.3)	**<0.0001**
Asthma/Reactive Airway Disease	213 (17.2)	111 (15.1)	0.231	152 (17.2)	103 (16.1)	0.574	69 (15.5)	0.435
Blood Disorder (e.g., Sickle cell disease)	191 (15.4)	84 (11.4)	**0.014**	96 (10.8)	126 (19.7)	**<0.0001**	53 (11.9)	0.567
Cancer	231 (18.6)	63 (8.6)	**<0.0001**	86 (9.7)	138 (21.5)	**<0.0001**	70 (15.7)	**0.001**
Cardiac Disease	906 (73.1)	447 (60.9)	**<0.0001**	502 (56.7)	488 (76.1)	**<0.0001**	363 (81.4)	**<0.0001**
Arrythmia	204 (16.5)	78 (10.6)	**0.0003**	89 (10.0)	110 (17.2)	**<0.0001**	83 (18.6)	**<0.0001**
Coronary Artery Disease	197 (15.9)	86 (11.7)	**0.010**	90 (10.2)	101 (15.8)	**0.0011**	92 (20.6)	**<0.0001**
Congestive Heart Failure (CHF)	356 (28.7)	134 (18.3)	**<0.0001**	180 (20.3)	168 (26.2)	**<0.0001**	142 (31.8)	**<0.0001**
Hypertension	821 (66.3)	395 (53.8)	**<0.0001**	450 (50.8)	434 (67.7)	**<0.0001**	332 (74.4)	**<0.0001**
Peripheral Vascular Disease (PVD)	50 (4.0)	19 (2.6)	0.091	19 (2.1)	22 (3.4)	0.124	28 (6.3)	**<0.0001**
Chronic Kidney Disease	301 (24.3)	155 (21.1)	0.106	147 (16.6)	172 (26.8)	**<0.0001**	137 (30.7)	**<0.0001**
Chronic Liver Disease	21 (1.7)	9 (1.2)	0.453	12 (1.4)	6 (0.9)	0.632	12 (2.7)	0.084
Cystic Fibrosis	13 (1.0)	3 (0.4)	0.193	5 (0.6)	6 (0.9)	0.542	5 (1.1)	0.317
Dementia	37 (3.0)	21 (2.9)	0.874	12 (1.4)	18 (2.8)	0.043	28 (6.3)	**<0.0001**
Diabetes Mellitus	390 (31.5)	203 (27.7)	0.074	224 (25.3)	203 (31.7)	**0.006**	166 (37.2)	**<0.0001**
HIV/AIDS	58 (4.7)	25 (3.4)	0.173	51 (5.8)	22 (3.4)	0.036	10 (2.2)	**0.004**
Seizure Disorder	45 (3.6)	19 (2.6)	0.206	21 (2.4)	22 (3.4)	0.216	21 (4.7)	**0.021**
Splenectomy	1 (0.1)	0 (0.0)	1.00	0 (0.0)	0 (0.0)	1.00	1 (0.2)	0.335
Any immunosuppressive condition (incl. cancer, HIV)	451 (36.4)	189 (25.7)	**<0.0001**	254 (28.7)	262 (40.9)	**<0.0001**	124 (27.8)	0.741
Strokes	121 (9.8)	49 (6.7)	**0.018**	56 (6.3)	60 (9.4)	0.027	54 (12.1)	**0.0003**
Currently taking steroids	146 (11.8)	88 (12.0)	0.892	100 (11.3)	105 (16.4)	**0.0004**	29 (6.5)	**0.005**
None	70 (5.7)	134 (18.3)	**<0.0001**	110 (12.4)	20 (3.1)	**<0.0001**	11 (2.5)	**<0.0001**
**Pregnant, No. (%)**	21 (1.7)	23 (3.1)	**0.036**	32 (3.6)	11 (1.7)	0.027	1 (0.2)	**0.0002**
**Charlson Comorbidity Indices ^b^, median (IQR)**								
Updated (uCCI) (range, 0–14)	1 [0, 3]	1 [0, 2]	**<0.0001**	1 [0, 2]	1 [0, 3]	**<0.0001**	2 [1, 3]	**<0.0001**
Classical (cCCI) (range, 0–15)	2 [1, 3]	1 [0, 2]	**<0.0001**	1 [0, 3]	2 [1, 3]	**<0.0001**	2 [1, 3]	**<0.0001**
**Prior SARS-CoV-2 Infection, No. (%)**			**<0.0001**			0.237		0.389
Yes	207 (16.7)	68 (9.3)		114 (12.9)	96 (15.0)		65 (14.6)	
No	1032 (83.3)	666 (90.7)		772 (87.1)	545 (85.0)		381 (85.4)	
**SARS-CoV-2 Positive, No. (%)**								
Yes	N/A	N/A		463 (52.3)	185 (28.9)		86 (19.3)	
No	N/A	N/A		423 (47.7)	456 (71.1)		360 (80.7)	
**COVID-19 Vaccination Status, No. (%)**								
Never Vaccinated for COVID-19	423 (34.1)	463 (63.1)		N/A	N/A		N/A	
Primary Series	456 (36.8)	185 (25.2)		N/A	N/A		N/A	
≥1 Booster Dose	360 (29.1)	86 (11.7)		N/A	N/A		N/A	
**Hospital History, No. (%)**								
Admitted to ICU	316 (25.5)	160 (21.8)	0.063	203 (22.9)	166 (25.9)	0.179	107 (24.0)	0.66
CPAP/BIPAP Use	143 (11.5)	63 (8.6)	**0.038**	83 (9.4)	74 (11.5)	0.167	49 (11.0)	0.351
Definite/Probable Pneumonia	585 (47.2)	438 (59.7)	**<0.0001**	516 (58.2)	337 (52.6)	0.028	170 (38.1)	**<0.0001**
Hospital Stay 4+ days	622 (50.2)	403 (54.9)	**0.043**	489 (55.2)	317 (49.5)	0.027	219 (49.1)	0.036
Mechanical Ventilation	78 (6.3)	42 (5.7)	0.607	67 (7.6)	31 (4.8)	0.032	22 (4.9)	0.070
**Discharge Location, No. (%)**			0.236			0.029		**0.0002**
Discharged to home	1077 (86.9)	636 (86.6)		773 (87.2)	567 (88.5)		373 (83.6)	
Discharged to Long-Term Care Facility	54 (4.4)	36 (4.9)		32 (3.6)	23 (3.6)		35 (7.8)	
Discharged to another hospital	10 (0.8)	5 (0.7)		7 (0.8)	3 (0.5)		5 (1.1)	
Discharged to hospice	44 (3.6)	15 (2.0)		17 (1.9)	24 (3.7)		18 (4)	
Death	24 (1.9)	23 (3.1)		23 (2.6)	14 (2.2)		10 (2.2)	
Other/Unknown	30 (2.4)	19 (2.6)		34 (3.8)	10 (1.6)		5 (1.1)	

* For comparisons of cases vs. controls, *p*-values ≤ 0.05 were considered statistically significant. ** For comparisons of unvaccinated vs. primary series or vs. booster series, Bonferroni correction was applied due to multiple bivariate comparisons, and *p*-values ≤ 0.025 were considered statistically significant. ^a^ Based on standard definitions for BMI classifications (<18.5 = underweight; 18.5 to <25 = healthy; 25 to <30 = overweight; 30+ = obese). ^b^ The Charlson Comorbidity Index predicts the 10-year mortality risk for a patient who may have multiple comorbid conditions; each of the 22 conditions (e.g., heart disease, cancer) is assigned a numerical weight depending on its associated risk, and weights are summed to provide an overall score. Bold *p*-values denote statistical significance.

**Table 4:** Crude and Adjusted Vaccine Effectiveness Estimates by outcome and time since most recent dose of mRNA COVID-19 vaccine.

VE Within Patients:	Pre-Delta/Delta Era (2 May 2021–19 December 2021)		Omicron Era (20 December 2021–31 January 2023)	
	Unadjusted (95% CI)	Adjusted (95% CI)	Unadjusted (95% CI)	Adjusted (95% CI)
**Overall**				
**No Vaccine vs. Primary Series**	82.6 (73.9, 88.4)	85.5 (77.0, 90.8)	24.1 (0.0, 42.4)	38.2 (11.5, 56.8)
<6 months since completion	86.2 (77.5, 91.6)	88.4 (79.9, 93.3)	8.2 (−42.1, 40.7)	65.5 (37.1, 81.1)
≥6 months since completion	73.2 (51.0, 85.3)	77.1 (54.4, 88.5)	28.6 (3.8, 47.0)	27.7 (−7.1, 51.2)
**No Vaccine vs. ≥1 Booster Dose**	95.0 (59.4, 99.4)	96.4 (68.9, 99.6)	53.9 (37.4, 66.0)	48.5 (20.8, 66.5)
<6 months since completion	94.2 (52.8, 99.3)	96.0 (64.7, 99.5)	58.8 (39.8, 71.8)	62.4 (37.1, 77.6)
≥6 months since completion	--	--	47.1 (21.3, 64.4)	8.8 (−63.3, 49.1)
**Definite/Probable Pneumonia**				
**No Vaccine vs. Primary Series**	88.3 (81.1, 92.8)	90.0 (82.6, 94.2)	37.6 (4.9, 59.0)	42.9 (2.3, 66.7)
<6 months since completion	90.7 (83.4, 94.8)	91.5 (83.8, 95.5)	31.4 (−27.6, 63.1)	71.1 (30.1, 88.1)
≥6 months since completion	81.9 (62.6, 91.2)	85.2 (65.9, 93.6)	40.0 (4.5, 62.3)	30.4 (−27.8, 62.1)
**No Vaccine vs. ≥1 Booster Dose**	--	--	56.2 (29.0, 72.9)	54.4 (9.0, 77.1)
<6 months since completion	--	--	67.5 (39.3, 82.6)	71.2 (33.4, 87.5)
≥6 months since completion	--	--	38.1 (−14.3, 66.5)	−44.3 (−300.1, 48.0)
**Hospital Stay ≥ 4 Days**				
**No Vaccine vs. Primary Series**	80.6 (65.7, 89.0)	83.6 (68.4, 91.5)	27.8 (−5.7, 50.6)	49.7 (16.3, 69.8)
<6 months since completion	82.4 (65.9, 90.9)	84.2 (66.9, 92.4)	1.4 (−79.8, 45.9)	71.9 (33.4, 88.2)
≥6 months since completion	76.0 (40.1, 90.4)	83.2 (53.8, 93.9)	34.9 (1.3, 57.1)	37.8 (−6.9, 63.9)
**No Vaccine vs. ≥1 Booster Dose**	--	--	57.9 (35.2, 72.6)	57.4 (20.6, 77.2)
<6 months since completion	--	--	61.8 (35.3, 77.5)	68.6 (33.7, 85.2)
≥6 months since completion	--	--	51.8 (14.0, 73.0)	12.3 (−110.1, 63.4)
**ICU Admission**				
**No Vaccine vs. Primary Series**	90.1 (72.6, 96.4)	94.4 (80.5, 98.4)	41.3 (−6.2, 67.5)	62.5 (19.0, 82.7)
<6 months since completion	90.4 (67.8, 97.1)	91.6 (67.1, 97.8)	15.0 (−104.6, 64.7)	75.6 (13.5, 93.1)
≥6 months since completion	89.6 (45.1, 98.0)	97.8 (82.9, 99.7)	48.5 (1.5, 73.1)	60.5 (6.9, 83.4)
**No Vaccine vs. ≥1 Booster Dose**	--	--	62.6 (25.1, 81.3)	48.7 (−40.9, 81.4)
<6 months since completion	--	--	54.7 (−1.2, 79.7)	66.1 (−20.3, 90.5)
≥6 months since completion	--	--	72.3 (23.1, 90.1)	−36.5 (−522.2, 70.1)

-- 0/low vaccinated counts; VE adjusted for age, enrollment quarter, sex, race/ethnicity, immunocompromised status, and enrollment site. Hybrid immunity VE estimates among participants with mechanical ventilation and death were not evaluable due to small numbers.

**Table 5:** Unadjusted and Adjusted Hybrid Vaccine Effectiveness.

VE Within Patients:	Pre-Delta/Delta Era (2 May 2021–19 December 2021)		Omicron Era (20 December 2021–31 January 2023)	
	Unadjusted (95% CI)	Adjusted (95% CI)	Unadjusted (95% CI)	Adjusted (95% CI)
**Overall**				
No Vaccine vs. Primary Series	91.4 (59.7, 98.2)	92.8 (63.5, 98.6)	54.0 (19.4, 73.8)	46.3 (−18.6, 75.7)
No Vaccine vs. ≥1 Booster Dose	--	--	55.5 (15.1, 76.7)	23.9 (−83.2, 68.4)
**Definite/Probable Pneumonia**				
No Vaccine vs. Primary Series	92.1 (62.2, 98.4)	92.7 (60.6, 98.6)	58.5 (4.1, 82.0)	29.3 (−133.4, 78.6)
No Vaccine vs. ≥1 Booster Dose	--	--	53.8 (−30.5, 83.7)	16.2 (−247.8, 79.8)
**Hospital Stay ≥4 Days**				
No Vaccine vs. Primary Series *	69.7 (−392.5, 98.1)	74.1 (−431.7, 98.7)	61.7 (13.5, 83.1)	64.0 (−31.0, 90.1)
No Vaccine vs. ≥1 Booster Dose	--	--	38.4 (−44.6, 73.8)	2.0 (−255.8, 73.0)
**ICU Admission**				
No Vaccine vs. Primary Series	--	--	69.7 (−10.1, 91.7)	-- (--, --) **
No Vaccine vs. ≥1 Booster Dose	--	--	61.3 (−85.6, 91.9)	-- (--, --) **

-- 0/low vaccinated counts; * 2 vaccinated in Pre/Delta; ** Small/missing numbers, cannot adjust. Vaccinated participants who reported previous SARS-CoV-2 infection compared to unvaccinated participants with no history of SARS-CoV-2 infection. VE adjusted for age, enrollment quarter, sex, race/ethnicity, immunocompromised status, and enrollment site. Hybrid Immunity VE estimates among participants with mechanical ventilation and death were not evaluable due to small numbers.

## Data Availability

Data will be made available upon reasonable request.

## Supplementary Materials

The following supporting information can be downloaded at: https://www.mdpi.com/article/10.3390/vaccines14010045/s1, Table S1: Baseline demographic characteristics of enrolled and non-enrolled patients; Table S2: Distribution of homologous and heterologous vaccine schedules among cases and controls; Table S3: Distribution of other respiratory pathogens detected among cases and controls; Table S4: Crude and adjusted vaccine effectiveness estimates by age category; Table S5: Crude and adjusted vaccine effectiveness estimates; Tables S6–S8: COVID VE sample size calculations.

## Abbreviations

The following abbreviations are used in this manuscript:

VE	Vaccine Effectiveness
US	United States
EUA	Emergency Use Authorization
EUH	Emory University Hospital
EUHM	Emory University Hospital Midtown
ARI	Acute Respiratory Infection
NP	Nasopharyngeal
SOC	Standard-of-Care
URI	Upper Respiratory Infection
EMR	Electronic Medical Record
GRITS	Georgia Immunization Registry
HIV	Human Immunodeficiency Virus
AIDS	Acquired Immune Deficiency Syndrome
RVP	Respiratory Viral Panel
OP	Oropharyngeal
IQR	Interquartile Range
CPAP	Continuous Positive Airway Pressure
BIPAP	Bilevel Positive Airway Pressure
BMI	Body Mass Index
ICU	Intensive Care Unit
