# Supplementary material for: mRNA COVID-19 Vaccine Effectiveness Against Severe Outcomes Among Adults Hospitalized with COVID-19 from May 2021 to January 2023
Source: Vaccines (Basel). 2025 Dec 30;14(1):45. doi: 10.3390/vaccines14010045 (PMC12846602; doi:10.3390/vaccines14010045)
Supplement: Supplementary file 1 [file vaccines-14-00045-s001.zip › vaccines-4008638-supplementary.pdf]

Supplementary Material for

mRNA COVID-19 Vaccine Effectiveness Against Severe Outcomes among Adults Hospitalized With COVID-19 from May 2021 to January 2023

Gabriella Ess, Ashley M. Lew, Ashley Tippett, Luis W. Salazar, Chris Choi, Khalel De Castro, Elizabeth G. Taylor, Olivia D. Reese, Humerazehra Momin, Caroline R. Ciric, Amrita Banerjee, Amy Keane, Laura A. Puzniak, Robin Hubler, Srinivas Valluri, Benjamin Lopman, Nadine Rouphael, Satoshi Kamidani, John M. McLaughlin, Evan J. Anderson and Christina A. Rostad

Supplementary Table S1. Baseline demographic characteristics of enrolled and non-enrolled patients.

	Not Enrolled (n=2378)*	Enrolled – Excluded (n=790)	Enrolled – Included (n=1973)
**Age, years (median [IQR])**	64 [48, 75]	59 [45, 69]	60 [48, 71]
**Sex****			
**Female**	1289 (54.2%)	403 (51.0%)	1105 (56.0%)
**Male**	1088 (45.8%)	387 (49.0%)	868 (44%)
**Ethnicity**			
**Hispanic**	60 (2.5%)	33 (4.2%)	49 (2.5%)
**Non-Hispanic**	2110 (88.7%)	696 (88.1%)	1789 (90.7%)
**Unknown**	208 (8.8%)	61 (7.7%)	135 (6.8%)
**Race**			
**American Indian, Alaskan Native**	6 (0.3%)	2 (0.3%)	1 (0.1%)
**Asian**	47 (2.0%)	18 (2.3%)	27 (1.4%)
**Black/AA**	1590 (66.9%)	537 (68.0%)	1343 (68.1%)
**Native Hawaiian or Pacific Islander**	2 (0.1%)	0 (0.0%)	1 (0.1%)
**White**	610 (25.6%)	194 (24.6%)	523 (26.5%)
**Unknown**	123 (5.2%)	39 (4.9%)	78 (4.0%)

*For this table, data was collected from the electronic medical record (EMR) for each group, as participant interview data was not available for those who did not enroll. For those who enrolled, there were minor differences in the demographic characteristics documented in the EMR vs what was self-reported in the participant interview.
**Data on sex was not available for one not enrolled patient.

Supplementary Table S2. Distribution of homologous and heterologous vaccine schedules among cases and controls.

	Total vaccinated (n=1087)	Primary series (n=641)	≥ 1 Booster Dose (n=446)	Controls (n=816)	Cases (n=271)
**Homologous Vaccine Series***	1020 (94%)	626 (98%)	394 (88%)	759 (93%)	261 (96%)
**Heterologous Vaccine Series**	67 (6%)	15 (2%)	52 (12%)	57 (7%)	10 (4%)

*Homologous vaccine series represents study participants who received all vaccine doses from the same manufacturer. Heterologous vaccine series represents those who received one or more doses from different vaccine manufacturers or whose vaccine manufacturer was unknown.

Supplementary Table S3. Distribution of other respiratory pathogens detected among cases and controls.

	Total (n=1973)		Controls (n=1239)		Cases (n=734)	
	Total	Total w/ Testing*	Controls Positive for other pathogens	Controls w/ Testing*	Cases Positive for other pathogens	Cases w/ Testing*
**Adenovirus**	0	308	0	240	0	68
**HMPV**	8 (3%)	308	8 (3%)	240	0	68
**Influenza**	95 (7%)	1415	90 (9%)	1014	5 (1%)	401
**Parainfluenza**	7 (2%)	308	7 (3%)	240	0	68
**Para 1**	0	308	0	240	0	68
**Para 2**	2 (1%)	308	2 (1%)	240	0	68
**Para 3**	5 (2%)	308	5 (2%)	240	0	68
**Rhino/Enterovirus**	25 (8%)	308	22 (9%)	240	3 (4%)	68
**RSV**	30 (2%)	1415	29 (3%)	1014	1 (<1%)	401

*Data represents those who had pathogen-specific testing performed per standard-of-care. HMPV, human metapneumovirus. Para 1, parainfluenza 3 virus; Para 2, parainfluenza 2 virus; Para 3, parainfluenza 3 virus. Rhino/enterovirus, rhinovirus/enterovirus (unable to distinguish on multiplexed panel); RSV, respiratory syncytial virus.

Supplementary Table S4. Crude and Adjusted Vaccine Effectiveness Estimates by Age category

VE within Patients:	Pre/Delta Era (May 2, 2021-Dec 19, 2021)		Omicron Era (Dec 20, 2021-Jan 31, 2023)	
**Hospitalized (All participants)**	Unadjusted (95% CI)	Adjusted (95% CI)	Unadjusted (95% CI)	Adjusted (95% CI)
No Vaccine vs. Primary Series				
18 to 64 years old	83.1 (70.7, 90.2)	85.4 (74.0, 91.8)	15.7 (-19.1, 40.3)	25.8 (-14.4, 51.9)
≥65 years old	81.5 (63.2, 90.8)	91.9 (80.3, 96.6)	48.9 (16.5, 68.7)	54.7 (14.0, 76.1)
No Vaccine vs. ≥1 Booster Dose				
18 to 64 years old	80.0 (-122.6, 98.2)	82.9 (-106.1, 98.6)	46.3 (16.5, 65.5)	16.7 (-45.4, 52.3)
≥65 years old	--	--	70.3 (51.3, 81.9)	70 (42.7, 84.6)
**Definite/Probable Pneumonia**	Unadjusted (95% CI)	Adjusted (95% CI)	Unadjusted (95% CI)	Adjusted (95% CI)
No Vaccine vs. Primary Series				
18 to 64 years old	89.4 (79.3, 94.6)	91.2 (82.2, 95.7)	39.2 (-6.0, 65.2)	37.4 (-29.5, 69.7)
≥65 years old	85.9 (68.1, 93.7)	92.2 (78.8, 97.1)	46.2 (-7.6, 73.1)	54.2 (-7.0, 80.4)
No Vaccine vs. ≥1 Booster Dose				
18 to 64 years old	--	--	54.8 (-1.1, 79.8)	-35.3 (-347.8, 59.1)
≥65 years old	--	--	66.0 (30.6, 83.3)	80.3 (45.6, 92.9)
**Hospital Stay ≥4 Days**	Unadjusted (95% CI)	Adjusted (95% CI)	Unadjusted (95% CI)	Adjusted (95% CI)
No Vaccine vs. Primary Series				
18 to 64 years old	80.8 (57.8, 91.3)	83.5 (61.8, 92.9)	25.5 (-24.0, 55.2)	37.2 (-21.3, 67.5)
≥65 years old	80.3 (49.9, 92.2)	88.4 (63.7, 96.3)	48.2 (4.4, 72.0)	61.7 (13.3, 83.1)
No Vaccine vs. ≥1 Booster Dose				
18 to 64 years old	--	--	60.2 (18.7, 80.5)	37.0 (-57.0, 74.7)
≥65 years old	--	--	72.0 (47.7, 85.0)	76.6 (42.5, 90.5)
**ICU Admission**	Unadjusted (95% CI)	Adjusted (95% CI)	Unadjusted (95% CI)	Adjusted (95% CI)
No Vaccine vs. Primary Series				
18 to 64 years old	92.1 (68.5, 98.0)	97.0 (82.9, 99.5)	55.3 (-1.2, 80.3)	80.7 (38.4, 94.0)
≥65 years old	85.4 (28.6, 97.0)	90.4 (30.9, 98.7)	37.3 (-66.8, 76.4)	49.1 (-100.0, 87.0)
No Vaccine vs. ≥1 Booster Dose				
18 to 64 years old	--	--	42.2 (-51.8, 78.0)	-2.7 (-319.4, 74.9)
≥65 years old	--	--	76.7 (30.0, 92.2)	89.3 (24.1, 98.5)
**Mechanical Ventilation**	Unadjusted (95% CI)	Adjusted (95% CI)	Unadjusted (95% CI)	Adjusted (95% CI)
No Vaccine vs. Primary Series				
18 to 64 years old	92.1 (-16.5, 99.5)	-- (--, --)**	23.3 (-372.1, 87.5)	71.7 (-242.2, 97.7)
≥65 years old	--	--	83.3 (-156.3, 98.9)	-- (--, --)**
No Vaccine vs. ≥1 Booster Dose				
18 to 64 years old	--	--	--	--

≥65 years old	--	--	65.4 (-208.0, 96.1)	-- (--, --)**
**Where Outcome = Death**	Unadjusted (95% CI)	Adjusted (95% CI)	Unadjusted (95% CI)	Adjusted (95% CI)
No Vaccine vs. Primary Series				
18 to 64 years old	--	--	70.0 (-390.8, 98.2)	-- (--, --)**
≥65 years old	75.0 (-756.0, 99.3)	-- (--, --)**	-200.0 (-4696.2, 81.2)	-- (--, --)**
No Vaccine vs. ≥1 Booster Dose				
18 to 64 years old	--	--	--	--
≥65 years old	--	--	--	--

-- 0/low vaccinated counts; ** Small/missing numbers, cannot adjust
VE adjusted for enrollment quarter, sex, race/ethnicity, immunocompromised status, and enrollment site.

Supplementary Table S5. Crude and Adjusted Vaccine Effectiveness Estimates

VE within Patients:	Pre-Delta/Delta Era (May 2, 2021-Dec 19, 2021)		Omicron Era (Dec 20, 2021-Jan 31, 2023)	
**Mechanical Ventilation**	Unadjusted (95% CI)	Adjusted (95% CI)	Unadjusted (95% CI)	Adjusted (95% CI)
No Vaccine vs. Primary Series	75.0 (-109.9, 97.0)	88.1 (-152.9, 99.4)	45.8 (-141.0, 87.8)	52.9 (-195.2, 92.5)
<6 months since completion	75.0 (-351.1, 98.6)	68.5 (-1014.5, 99.1)	-73.3 (-1018.7, 73.1)	10.6 (-1327.2, 94.4)
≥6 months since completion	75.0 (-351.1, 98.6)	99.5 (19.1, 99.9)	77.2 (-105.4, 97.5)	77.5 (-220.1, 98.4)
No Vaccine vs. ≥1 Booster Dose	--	--	31.6 (-208.8, 84.8)	36.7 (-983.2, 96.3)
<6 months since completion	--	--	33.3 (-277.3, 88.2)	--(--, --)*
≥6 months since completion	--	--	27.8 (-617.2, 92.7)	--(--, --)*
**Where Outcome = Death**	Unadjusted (95% CI)	Adjusted (95% CI)	Unadjusted (95% CI)	Adjusted (95% CI)
No Vaccine vs. Primary Series	92.3 (-139.4, 99.7)	-- (--, --)*	0 (-525.5, 84.0)	--(--, --)*
<6 months since completion	--	--	-100.0 (-2105.6, 81.9)	--(--, --)*
≥6 months since completion	92.3 (-139.4, 99.7)	-- (--, --)*	33.3 (-453.7, 92.0)	--(--, --)*
No Vaccine vs. ≥1 Booster Dose	--	--	50.0 (-299.8, 93.7)	--(--, --)*
<6 months since completion	--	--	50.0 (-568.3, 96.3)	--(--, --)*
≥6 months since completion	--	--	50.0 (-568.3, 96.3)	--(--, --)*

-- 0/low vaccinated counts; *small/missing numbers, cannot adjust
VE adjusted for age, enrollment quarter, sex, race/ethnicity, immunocompromised status, and enrollment site.

Supplementary Tables S6–S8. COVID VE Sample Size Calculations

Requirements for the final analysis population to detect mRNA VE >20% assuming true VE=70–90% with 90% power and type-I error of 5% (2-sided) under various uptake scenarios (5 to 30% of ARI hospitalizations due to SARS-CoV-2). ARI, acute respiratory illness; VE, vaccine effectiveness.

**Assume true VE=70%**																
	5% of ARI is SARS-CoV-2 positive				15% of ARI is SARS-CoV-2 positive				25% of ARI is SARS-CoV-2 positive				30% of ARI is SARS-CoV-2 positive			
Uptake	Controls	Cases	Tot Eval	Tot Enroll*	Controls	Cases	Tot Eval	Tot Enroll*	Controls	Cases	Tot Eval	Tot Enroll*	Controls	Cases	Tot Eval	Tot Enroll*
10	6769	356	7125	11875	2104	371	2475	4125	1171	390	1561	2602	938	402	1340	2233
20	3266	172	3438	5730	1022	180	1202	2003	573	191	764	1273	461	198	659	1098
30	2108	111	2219	3698	665	117	782	1303	377	126	503	838	304	130	434	723
40	1540	81	1621	2702	491	87	578	963	281	94	375	625	229	98	327	545
50	1213	64	1277	2128	392	69	461	768	228	76	304	507	187	80	267	445
60	1015	53	1068	1780	335	59	394	657	199	66	265	442	165	71	236	393
70	909	48	957	1595	308	54	362	603	187	62	249	415	157	67	224	373
80	905	48	953	1588	318	56	374	623	200	67	267	445	171	73	244	407
90	1174	62	1236	2060	435	77	512	853	287	96	383	638	251	108	359	598
**Assume true VE=80%**																
	5% of ARI is SARS-CoV-2 positive				15% of ARI is SARS-CoV-2 positive				25% of ARI is SARS-CoV-2 positive				30% of ARI is SARS-CoV-2 positive			
Uptake	Controls	Cases	Tot Eval	Tot Enroll*	Controls	Cases	Tot Eval	Tot Enroll*	Controls	Cases	Tot Eval	Tot Enroll*	Controls	Cases	Tot Eval	Tot Enroll*
10	4945	260	5205	8675	1518	268	1786	2977	832	277	1109	1848	661	283	944	1573
20	2325	122	2447	4078	717	127	844	1407	396	132	528	880	315	135	450	750
30	1455	77	1532	2553	452	80	532	887	252	84	336	560	201	86	287	478
40	1024	54	1078	1797	321	57	378	630	181	60	241	402	146	63	209	348
50	770	41	811	1352	245	43	288	480	140	47	187	312	114	49	163	272
60	608	32	640	1067	197	35	232	387	115	38	153	255	95	41	136	227

70	505	27	532	887	169	30	199	332	102	34	136	227	85	36	121	202
80	455	24	479	798	160	28	188	313	101	34	135	225	86	37	123	205
90	513	27	540	900	196	35	231	385	132	44	176	293	116	50	166	277
**Assume true VE=90%**																
	5% of ARI is SARS-CoV-2 positive				15% of ARI is SARS-CoV-2 positive				25% of ARI is SARS-CoV-2 positive				30% of ARI is SARS-CoV-2 positive			
Uptake	Controls	Cases	Tot Eval	Tot Enroll*	Controls	Cases	Tot Eval	Tot Enroll*	Controls	Cases	Tot Eval	Tot Enroll*	Controls	Cases	Tot Eval	Tot Enroll*
10	4275	225	4500	7500	1294	228	1522	2537	698	233	931	1552	549	235	784	1307
20	1955	103	2058	3430	594	105	699	1165	322	107	429	715	253	108	361	602
30	1183	62	1245	2075	361	64	425	708	197	66	263	438	155	66	221	368
40	798	42	840	1400	245	43	288	480	135	45	180	300	107	46	153	255
50	568	30	598	997	176	31	207	345	98	33	131	218	78	33	111	185
60	417	22	439	732	132	23	155	258	74	25	99	165	60	26	86	143
70	313	16	329	548	101	18	119	198	59	20	79	132	49	21	70	117
80	241	13	254	423	83	15	98	163	51	17	68	113	43	18	61	102
90	212	11	223	372	82	14	96	160	56	19	75	125	50	21	71	118
